# The long-term association of different dietary protein sources with metabolic syndrome

**DOI:** 10.1038/s41598-021-98688-0

**Published:** 2021-09-29

**Authors:** Parisa Hajihashemi, Razieh Hassannejad, Fahimeh Haghighatdoost, Noushin Mohammadifard, Masoumeh Sadeghi, Hamidreza Roohafza, Firoozeh Sajjadi, Nizal Sarrafzadegan

**Affiliations:** 1 Hypertension Research Center, Cardiovascular Research Institute, Isfahan University of Medical Sciences, Isfahan, Iran; 2 Heart Failure Research Center, Cardiovascular Research Institute, Isfahan University of Medical Sciences, Isfahan, Iran; 3 Isfahan Cardiovascular Research Center, Cardiovascular Research Institute, Isfahan University of Medical Sciences, PO Box 81745, Isfahan, Iran; 4 Cardiac Rehabilitation Research Center, Cardiovascular Research Institute, Isfahan University of Medical Sciences, Isfahan, Iran; 5 Interventional Cardiology Research Center, Cardiovascular Research Institute, Isfahan University of Medical Sciences, Isfahan, Iran

**Keywords:** Cardiology, Diseases, Risk factors

## Abstract

Due to scarce epidemiologic data linking dietary protein intakes and metabolic syndrome (MetS), we aim to determine the longitudinal association of different types of dietary protein with the incidence of MetS among Iranians adults. The study was conducted in the framework of the Isfahan Cohort Study (ICS) on 6504 adults, aged ≥ 35 years, and free of MetS at baseline. A validated food frequency questionnaire was used for assessing usual dietary intakes. MetS was defined according to the Joint Scientific Statement. Mixed-effects logistic regression was applied to examine the associations between changes in weekly frequency consumption of protein and MetS status. After a median follow-up of 11.25 years, in multivariate-adjusted model, each additional frequency consumption of total protein intake (OR 0.83; 95% CI 0.81–0.85), animal protein (OR 0.80; 95% CI 0.77–0.83), plant protein (OR 0.70; 95% CI 0.64–0.76), red meat (OR 0.74; 95% CI 0.70–0.78), poultry (OR 0.73; 95% CI 0.68–0.78), egg (OR 0.79; 95% CI 0.72–0.88) and nuts and seeds (OR 0.77; 95% CI 0.71–0.84) was associated with reduced risk of MetS. No significant association was found for processed meat (OR 0.96; 95% CI 0.87–1.01) and legumes and soy (OR 0.96; 95% CI 0.86–1.07) with MetS. Our results suggest an independent inverse association between total protein, animal and plant protein and the risk of MetS. These associations did not differ by sex. Although our results can be considered to be a strategy to reduce MetS risk by dietary guidelines, randomized clinical trials are required to confirm our findings.

## Introduction

Metabolic syndrome (MetS) is characterized by clustering of at least three of the five following abnormalities: impaired fasting glucose, central obesity, low high density lipoprotein (HDL-C), elevated triglyceride (TG) levels, and hypertension^1^. These medical conditions increase the risk of cardiovascular disease (CVD), type II diabetes, and all-cause mortality^2^. MetS become a public health problem in both developed and developing countries^3^. The prevalence of MetS has been growing rapidly worldwide in the last decades^3^. It is estimated that at least 30% of Iranian adults are affected by MetS^4^.

Lifestyle modifications including physical activity, weight loss and dietary change play crucial role in the management of MetS^5^. Although food groups and dietary patterns have been frequently investigated in relation to MetS^6–8^, there is sparse information on dietary protein intake and its types in this regard.

High protein diet may decrease risk of various chronic diseases via its beneficial effects on body weight/composition, dyslipidemia, glucose intolerance and hypertension^9^. However, the effects of dietary protein intake on metabolic parameters may be influenced by the source of protein^10^. Several studies have suggested that a diet high in animal protein is associated with an increased risk of CVD and MetS^10–15^. In contrast, consumption of plant-based protein was inversely related to MetS components^10,16,17^. Similarly, a 11-year follow-up cohort study in Australian adults showed that protein from red meat and chicken was related to higher incidence of MetS, whereas protein from grains, legumes and nuts was associated with lower incident MetS^10^. Epidemiological evidence regarding the relation of animal protein intake and risk of chronic diseases remains mixed. Recent studies revealed that various types of animal protein may differently affect cardiometabolic risk factors. A systematic review revealed that plant-sourced protein, especially soy protein with isoflavones, were associated with a healthier cardiometabolic profile, that is, lower serum cholesterol levels and blood pressure. Nevertheless, no beneficiary association was found for glycemic parameters and anthropometric measures^17^. This discrepancy is also observed for different types of animal-sourced proteins. A recent cross-sectional study showed that red but not white meat consumption was associated with higher occurrence of MetS^18^. In terms of non-communicable chronic diseases, results are also inconsistent. In a 26-year follow-up prospective cohort study, higher consumption of red meat was associated with an increased risk of coronary heart disease (CHD). In contrast, this study suggested that higher consumption of dairy products, poultry, and fish be associated with a lower risk of coronary disease^19^.

Although the relation between different dietary protein types (including red meat, fish, dairy, nuts and legumes) and MetS components has been studied^10,15–17,20–22^, few longitudinal studies are available in this regard. Since macro nutrients contribution to daily energy intake and also their main sources may vary from a population to another one, their health outcomes might be also different. Therefore, we aimed to determine the longitudinal association of different types of dietary protein according to their originated from with the incidence of MetS among a large sample of Iranian adults in Isfahan Cohort Study (ICS). We hypothesized that dietary protein derived from different sources (animal or plant) would be differently related to the risk of incident MetS.

## Methods and materials

### Study population

This study was conducted in the framework of the ICS, a population-based longitudinal cohort study^23^. The ICS was established in 2001 and conducted in three districts of central Iran. A total of 6504 adults (3168 men and 3336 women) aged ≥ 35 years were recruited using stratified cluster random sampling method (2153 from Isfahan, 1028 from Najaf-Abad, and 3323 from Arak). Further detailed description about study design has been presented elsewhere^23^. At baseline, data on lifestyle factors including dietary intake, smoking status, physical activity and medical history (e.g. the history of dyslipidemia, diabetes mellitus, hypertension, and medicine use)^24,25^ were collected by trained health professionals using face-to-face 30-min home-interview and participants were followed up biannually. Physical activity was estimated using a validated questionnaire^26^. When no cardiovascular event occurred in annual evaluations, all variables measurements were repeated in the next six year of follow-up surveys (2007 and 2013). At the end, data from 1388 participants with no CVD event, who attended for repeated measurements in both 2007 and 2013 and had complete information on dietary intake and covariates, were included in our analysis. More investigation presented that there was no significant difference between those participants lost to follow-up compared with those remained in the study. All participants expressed their willingness to participate in the study through an informed written consent. All methods were performed in accordance with the relevant guidelines and regulations. This study was approved by the Ethics Committee of the Research Council of Isfahan Cardiovascular Research Center, a World Health Organization collaborating center in Isfahan, Iran.

### Anthropometric measurements

Height was measured to the nearest 0.5 cm using a nonelastic meter while the subject was barefoot and standing in a normal position^27^. Weight was measured on a scale to the nearest 100 g while subjects were in light clothing. Waist circumference (WC) was measured at a level midway between the lower rib margin and the iliac crest using a tape horizontally fixed around the body. Hip circumference was measured at the point of maximum circumference over the buttocks using a nonelastic meter^27^. Body mass index (BMI) was calculated as weight (kg) divided by height (m^2^). All measurements were performed in 2001 and repeated in 2007 and 2013.

### Definition of metabolic syndrome

MetS was defined based on the Joint Scientific Statement^28^. Accordingly, in order for someone to be considered to be affected by MetS, the presence of at least three of the following criteria was required. These factors included: (1) elevated fasting blood glucose (FBG ≥ 100 mg/dl) or current use of anti-diabetic medications, (2) elevated blood pressure (SBP ≥ 130 mmHg or DBP ≥ 85 mmHg) or current use of anti- hypertensive medications, (3) elevated serum TG level (TG ≥ 150 mg/dl) or current use of anti-dyslipidemic medications, (4) reduced HDL-C (HDL-C < 40 mg/dl in men and HDL-C < 50 mg/dl in women), and (5) abdominal obesity (WC ≥ 102 cm in men and ≥ 88 cm in women).

### Dietary assessment

A validated 48-item food frequency questionnaire (FFQ) was used to collect habitual dietary intake of participants in the preceding year at three phases^29,30^. Trained health professionals completed questionnaires through face-to-face interviews. Participants were asked to report the mean frequency of consumption of each food item during the past year in an open-ended format (daily, weekly, or monthly). They were also asked to opt “never/seldom”, which was considered to be zero, when a certain food item was never consumed or consumed less than once a month. We did not collect information about portion sizes of food items, however, it might not be main concern for our data, for the validation study of our FFQ showed that portion sizes were less likely to vary compared with the frequency of intake for most food items^30^.

Animal-sourced proteins contained egg, dairy products, red meat, poultry and fish. Dairy products intake included the frequency intake of high-fat milk, yogurt, and cheese. Red meat included all types of beef and lamb, and poultry included chicken and turkey. For fish intake, we did not consider canned fish, because its high sodium content could mislead the results. Plant-sourced proteins contained legumes and nuts. Legumes included both soy and non-soy legumes and nuts included seeds, pistachio, almond, hazelnut and walnut.

The FFQ was validated against a single 24-h recall and two food records. Significant correlations were found between the frequency consumption of animal protein, plant protein, and dairy products derived from FFQ and the intake estimated by dietary recall and records (Spearman’s rank correlation coefficient for: animal protein = 0.294; *p* = 0.007, plant protein = 0.480; *p* < 0.001, dairy products = 0.467; *p* < 0.001, nuts = 0.468; *p* < 0.001)^30^.

### Statistical analysis

Continuous and categorical variables were reported as mean ± SD and frequency (n, %), respectively. Non-normal continuous variables were presented as median (Q1–Q3)*.* Differences in quantitative variables across MetS status was assessed by using Student *t* test or Mann–Whitney *U* test (if the normality assumption was not held). In terms of categorical variables, we applied chi-square test to evaluate the distribution of them across MetS status. Analysis of covariance was applied to obtain age- and sex-adjusted mean intakes of protein sources across MetS status.

Mixed-effects logistic regression with coefficients presented on the logit scale was applied to evaluate the associations of changes in weekly frequency consumption of protein with MetS status. This model takes into account the normal random intercept as a random effect embedded within the linear predictor to consider the longitudinal nature of the data^31^. The models were adjusted for variables which were identified as confounders in literature. These included age, sex, smoking status, physical activity, protein sources other than the independent source and BMI. The sensitivity analysis was also performed based on stratification of sex.

The residual method was used to obtain energy-adjusted intake of protein sources^32^. Due to the lack of data on energy intake in this study and since energy intake cannot be an accurate measure of energy balance, we adjusted protein intakes for BMI as a surrogate measure^33^. Protein BMI-adjusted intake was considered in modeling process as a time-varying covariate. Statistical analyses were performed, using STATA software, version 14. *p* < 0.05 (two-tailed) was considered as statistically significant.

## Results

A total of 4163 individuals were included in our study. 1869 (45%) had MetS. The general characteristics of participants according to the presence or absence of MetS in the three years of data collection are shown in Table 1. In all 3 years (2001, 2007 and 2013) subjects with MetS were older and had lower physical activity levels, lower HDL-C, but higher BMI, WC, SBP, DBP, TC, LDL-C, TG and FBG. They were also less likely to be male, highly educated and smoker but more likely to have a history of hypertension, diabetes mellitus and dyslipidemia.

**Table 1:** General characteristics of participants according to the presence or absence of metabolic syndrome.

Variables	2001			2007			2013		
	Metabolic syndrome	No metabolic syndrome	*P* value^2^	Metabolic syndrome	No metabolic syndrome	*P* value^2^	Metabolic syndrome	No metabolic syndrome	P value^2^
Subjects (n)	531	857		555	833		783	604	
Age (years)	48.00 (42.00–56.00)	44.00 (40.00–51.00)	< 0.0001	48.00 (42.00–56.00)	44.00 (40.00–51.00)	< 0.0001	46.00 (41.00–53.00)	44.00 (39.00–52.00)	0.001
BMI (kg/m^2^)	29.81 ± 4.02	26.00 ± 4.14	< 0.0001	29.61 ± 4.05	26.50 ± 4.20	< 0.0001	29.63 ± 4.34	26.04 ± 4.24	< 0.0001
WC (cm)	104.63 ± 9.47	94.03 ± 10.90	< 0.0001	99.40 ± 9.23	90.47 ± 11.13	< 0.0001	102.20 ± 9.84	91.87 ± 10.37	< 0.0001
Male n (%)	159 (29.90)	515 (60.10)	< 0.0001	207 (37.30)	467 (56.10)	< 0.0001	313 (40.00)	361 (59.80)	< 0.0001
Physically activity level	11.00 (6.07–18.00)	15.00 (9.00–21.00)	< 0.0001	12.00 (8.00–17.10)	13.00 (9.00–18.00)	0.003	10.00 (6.00–15.00)	11.00 (7.00–17.00)	< 0.0001
**Educational level n (%)**									
0–5 years	368 (69.60)	453 (52.90)	< 0.0001	366 (66.10)	463 (55.70)	< 0.0001	498 (63.00)	315 (52.70)	< 0.0001
6–12 years	136 (25.70)	318 (37.10)	< 0.0001	150 (27.10)	276 (33.20)	< 0.0001	225 (29.00)	202 (33.80)	< 0.0001
> 12 years	25 (4.70)	86 (10.00)	< 0.0001	38 (6.90)	92 (11.10)	< 0.0001	62 (8.00)	81 (13.50)	< 0.0001
Current smokers *n* (%)	39 (7.40)	179 (20.90)	< 0.0001	56 (10.10)	125 (15.10)	0.007	82 (10.60)	103 (17.1)	< 0.0001
TC (mg/dl)	233.73 ± 50.98	211.68 ± 50.59	< 0.0001	218.32 ± 45.27	204.75 ± 39.69	< 0.0001	200.13 ± 42.21	199.66 ± 39.25	0.829
HDL (mg/dl)	43.00 (37.00–49.00)	48.00 (41.00–56.00)	< 0.0001	41.00 (36.00–48.00)	48.00 (41.00–56.00)	< 0.0001	40.00 (35.00–46.00)	46.00 (40.00–53.00)	< 0.0001
LDL (mg/dl)	140.85 ± 42.72	127.87 ± 41.66	< 0.0001	131.79 ± 31.74	124.50 ± 28.69	< 0.0001	111.50 ± 28.15	111.87 ± 27.14	0.808
Triglyceride (mg/dl)	214.00 (169.00–294.00)	144.00 (106.00–211.00)	< 0.0001	199.00 (155.00–280.00)	112.00 (80.00–154.00)	< 0.0001	167.00 (127.00–213.00)	110.00 (87.00–137.00)	< 0.0001
FBS (mg/dl)	87.00 (76.00–102.00)	78.00 (72.00–86.00)	< 0.0001	100.00 (89.00–122.00)	88.00 (82.00–93.00)	< 0.0001	104.00 (93.00–124.00)	91.00 (84.00–97.00)	< 0.0001
SBP	130.00 (113.00–140.00)	110.00 (103.00–120.00)	< 0.0001	130.00 (120.00–140.00)	120.00 (110.00–130.00)	< 0.0001	130.00 (120.00–143.00)	120.00 (115.00–130.00)	< 0.0001
DBP	80.00 (75.00–90.00)	73.00 (70.00–80.00)	< 0.0001	80.00 (80.00–87.00)	80 (70–80)	< 0.0001	85.00 (80.00–90.00)	80.00 (80.00–85.00)	< 0.0001
History of HTN n(%)	238 (44.80)	90 (10.50)	< 0.0001	288 (51.90)	169 (20.30)	< 0.0001	505 (64.50)	168 (27.90)	< 0.0001
History of Diabetes mellitus n(%)	77 (14.50)	19 (2.20)	< 0.0001	162 (29.20)	34 (4.10)	< 0.0001	254 (32.50)	29 (4.80)	< 0.0001
History of Dyslipidemia (%)	529 (99.60)	713 (83.20)	< 0.0001	550 (99.10)	626 (75.20)	< 0.0001	768 (98.10)	448 (74.40)	< 0.0001

Continuous variables were expressed as mean ± SD, non-normal continuous variables were expressed as median (Q1–Q3) and categorical variables were expressed as frequency and percent.

BMI, body mass index; WC, waist circumference; TC, total cholesterol; HDL, High-density lipoprotein cholesterol; LDL, Low-density lipoprotein cholesterol; TG, Triglyceride; FBS, Fasting blood sugar; SBP, Systolic blood pressure; DBP, Diastolic blood pressure; HTN; hypertension.

Dietary intakes of participants according to the presence or absence of MetS and the year of data collection are summarized in Table 2. Poultry, nuts, fruit and vegetables intake were consumed in greater amounts by the individuals with MetS in 2001. In 2007, non-hydrogenated vegetable oils intake was more frequent in participants with MetS. In 2007 and 2013, egg consumption was less frequent in participants with MetS. Fruits and vegetables consumption was more frequent in participants with MetS in 2013, whereas sweet beverages consumption was less frequent in participants with MetS in 2013.

**Table 2:** Dietary intakes of participants according to the presence or absence of metabolic syndrome.

Variables (frequency of consumption)	2001			2007			2013		
	Metabolic syndrome	No metabolic syndrome	*P* value	Metabolic syndrome	No metabolic syndrome	*P* value	Metabolic syndrome	No metabolic syndrome	*P* value
Subjects (n)	531	857		555	833		783	604	
Red meat	3.99 ± 0.12	3.98 ± 0.01	0.95	2.91 ± 0.10	2.90 ± 0.08	0.94	0.71 ± 0.07	0.72 ± 0.07	0.87
Processed meat	0.43 ± 0.04	0.38 ± 0.03	0.33	0.23 ± 0.02	0.20 ± 0.02	0.27	0.07 ± 0.01	0.09 ± 0.01	0.08
Poultry	2.05 ± 0.08	1.80 ± 0.06	0.02	2.22 ± 0.07	2.16 ± 0.06	0.53	0.18 ± 0.03	0.21 ± 0.04	0.55
Fish	0.51 ± 0.05	0.45 ± 0.03	0.24	0.80 ± 0.06	0.79 ± 0.05	0.91	0.97 ± 0.10	0.77 ± 0.11	0.19
Dairy	0.41 ± 0.08	0.54 ± 0.06	0.23	0.18 ± 0.04	0.22 ± 0.04	0.54	0.32 ± 0.05	0.41 ± 0.06	0.25
Egg	1.72 ± 0.08	1.77 ± 0.07	0.59	1.31 ± 0.05	1.45 ± 0.04	0.01	1.18 ± 0.04	1.35 ± 0.05	0.01
Nuts	1.29 ± 0.11	1.05 ± 0.09	0.09	1.38 ± 0.12	1.57 ± 0.10	0.25	0.53 ± 0.05	0.60 ± 0.06	0.42
Legumes	2.81 ± 0.10	2.97 ± 0.08	0.26	2.67 ± 0.08	2.58 ± 0.06	0.37	2.32 ± 0.06	2.35 ± 0.07	0.77
Animal protein	9.12 ± 0.21	8.93 ± 0.17	0.49	7.65 ± 0.17	7.72 ± 0.14	0.75	3.43 ± 0.15	3.56 ± 0.17	0.57
Plant protein	4.10 ± 0.16	4.01 ± 0.13	0.66	4.06 ± 0.15	4.15 ± 0.12	0.65	2.85 ± 0.08	2.94 ± 0.10	0.47
Total protein	13.22 ± 0.30	12.93 ± 0.24	0.47	11.70 ± 0.24	11.87 ± 0.20	0.61	6.28 ± 0.17	6.50 ± 0.20	0.41
Hydrogenated vegetable oils	6.17 ± 0.20	6.23 ± 0.16	0.84	2.07 ± 0.21	2.30 ± 0.17	0.39	1.35 ± 0.11	1.24 ± 0.13	0.50
Non-Hydrogenated vegetable oils	2.61 ± 0.19	2.74 ± 0.14	0.59	6.76 ± 0.17	6.20 ± 0.14	0.01	5.18 ± 0.14	5.38 ± 0.16	0.35
Fruits, vegetables	13.13 ± 0.32	12.22 ± 0.26	0.03	15.89 ± 0.33	16.23 ± 0.27	0.43	14.37 ± 0.27	12.84 ± 0.31	< 0.0001
Sweet beverages	3.81 ± 0.23	3.57 ± 0.18	0. 42	1.39 ± 0.10	1.50 ± 0.77	0.37	1.45 ± 0.09	1.99 ± 0.10	< 0.0001

Data are expressed as mean ± SE; all values are adjusted by age and sex (Obtained from ANCOVA).

Table 3 provides the crude and multivariate-adjusted ORs for MetS and frequency consumption of dietary protein intake. The frequency of total protein consumption was associated with 17% lower odds of MetS in the crude model (OR 0.83; 95% CI 0.81–0.85). Adjustment for possible confounders just slightly changed the associations (OR 0.84; 95% CI 0.81–0.87).

**Table 3:** The odds ratio for metabolic syndrome per each increment in the frequency of consumption of different protein source.

Variables	Crude	Model 1	Model 2	Model 3
Total protein	0.83 (0.81, 0.85)	0.83 (0.81, 0.86)	0.85 (0.82, 0.87)	0.84 (0.81, 0.87)
Animal protein	0.80 (0.77,0.83)	0.81 (0.78, 0.84)	0. 82(0.79, 0.85)	0.81 (0.78, 0.85)
Plant protein	0.70 (0.64, 0.76)	0.72 (0.67, 0.79)	0.75 (0.69, 0.82)	0.79 (0.72, 0.87)
Red meat	0.74 (0.70, 0.78)	0.75 (0.71, 0.79)	0.77 (0.73, 0.82)	0.81 (0.75, 0.87)
Processed meat	0.87 (0.79, 0.96)	0.92 (0.83, 1.00)	0.92 (0.84, 1.01)	0.96 (0.87, 1.07)
Poultry	0.73 (0.68, 0.78)	0.73 (0.68, 0.79)	0.76 (0.71, 0.82)	0.78 (0.72, 0.85)
Fish	1.10 (1.01, 1.20)	1.10 (1.02, 1.20)	1.01 (0.99, 1.12)	1.06 (0.96, 1.17)
Egg	0.79 (0.72, 0.88)	0.82 (0.75, 0.91)	0.83 (0.76, 0.92)	0.89 (0.80, 0.98)
Dairy	0.98 (0.89, 1.07)	0.98 (0.90, 1.07)	0.99 (0.91, 1.09)	1.03 (0.94, 1.14)
Nuts and seeds	0.77 (0.71, 0.84)	0.80 (0.73, 0.87)	0.81 (0.74, 0.89)	0.87 (0.78, 0.96)
Legumes, soy	0.82 (0.75, 0.90)	0.86 (0.78, 0.94)	0.88 (0.80, 0.96)	0.96 (0.86, 1.07)

Model 1: Adjusted for age (year) and sex (men/women).

Model 2: Additionally adjusted for physical activity, current smoker and BMI.

Model 3: Additionally adjusted for fruits and vegetables, cereal and protein sources.

In terms of animal protein, in the crude model, each additional increment in the frequency of consumption was associated with 20% decrease in the odds of MetS (OR 0.80; 95% CI 0.77–0.83). Controlling for possible confounders did not affect the association (OR 0.80; 95% CI 0.78–0.85). A significant inverse association was also observed between odds of MetS and frequency consumption of red meat (OR 0.74; 95% CI 0.70–0.78), poultry (OR 0.73; 95% CI 0.68–0.78), and egg (OR 0.79; 95% CI 0.72–0.88). When potential confounders were taken into account, the associations remained almost identical for red meat (OR 0.81; 95% CI 0.75–0.87), poultry (OR 0.78; 95% CI 0.72–0.85) and egg (OR 0.89; 95% CI 0.80–0.98). Regarding processed meat, in the crude model, each increment in the frequency of consumption was associated with lower odds of MetS. However, adjustment for potential confounders disappeared the significance (OR 0.96; 95% CI 0.87–1.01).

A significant inverse association was observed between frequency of plant protein consumption and odds of MetS (OR 0.70; 95% CI 0.64–0.76). Further adjustment for potential confounders just slightly weakened the association. In the crude model, the odds of MetS decreased by 23% per each additional frequency of nuts and seeds consumption (OR 0.77; 95% CI 0.71–0.84). Legumes and soy consumption was inversely associated with odds of MetS in the crude model (OR 0.82; 95% CI 0.75–0.90). However, after adjustment for potential confounders, the association was no longer significant (OR 0.96; 95% CI 0.86–1.07).

Table 4 provides the crude and multivariate-adjusted ORs for MetS and frequency of consumption of dietary protein intake stratified by sex. Similar to the whole population, in both men and women, significant inverse associations were observed between odds of MetS and frequency of total, animal and plant protein consumption. Adjustment for potential confounders did not alter the associations substantially. Regarding different protein sources, in the crude model, each increment in the frequency consumption of red meat, poultry, egg, nuts and seeds, and legumes and soy was associated with lower risk of MetS in both men and women. However, adjustment for covariates led to non-significant associations for egg in women and for nuts and seeds in men. The inverse association of legumes and soy with MetS remained no longer significant after adjustment for confounders either in men or in women. Regarding processed meat, significant inverse association was observed between odds of MetS and frequency of consumption in women, but not men, in the crude model. However, after adjustment for potential confounders, the association remained no longer significant. Fish and dairy products were pertinent to MetS neither in men nor in women.

**Table 4:** Odds ratio for metabolic syndrome per each increment in the frequency of consumption of different protein sources stratified by sex.

Variables	Crude		Multivariate-adjusted	
	Male	Female	Male	Female
Total protein	0.81 (0.78, 0.85)	0.85 (0.82, 0.88)	0.82 (0.78, 0.86)	0.85 (0.82, 0.89)
Animal protein	0.78 (0.74,0.82)	0.83 (0.79, 0.87)	0.78 (0.74, 0.83)	0.84 (0.79, 0.87)
Plant protein	0.72 (0.63, 0.81)	0.70 (0.22, 0.78)	0.82 (0.71, 0.94)	0.77 (0.68, 0.87)
Red meat	0.71 (0.65, 0.77)	0.77 (0.71, 0.82)	0.81 (0.73, 0.90)	0.80 (0.72, 0.89)
Processed meat	0.93 (0.82, 1.06)	0.83 (0.73, 0.96)	0.98 (0.85, 1.13)	0.92 (0. 78, 1.07)
Poultry	0.67 (0.60, 0.74)	0.79 (0.72, 0.87)	0.72 (0.63, 0.81)	0.83 (0.74, 0.94)
Fish	1.12 (0.99, 1.26)	1.10 (0.97, 1.24)	1.05 (0.92, 1.20)	1.09 (0.93, 1.26)
Egg	0.76 (0.66, 0.88)	0.82 (0.72, 0.93)	0.82 (0.70, 0.96)	0.94 (0.82, 1.07)
Dairy	1.00 (0.88, 1.14)	0.96 (0.85, 1.09)	1.06 (0.92, 1.22)	1.00 (0.87, 1.15)
Nuts and seeds	0.81 (0.70, 0.92)	0.75 (0.67, 0.84)	0.92 (0.79, 1.07)	0.82 (0.71, 0.94)
Legumes, soy	0.84 (0.74, 0.96)	0.82 (0.73, 0.93)	1.03 (0.89, 1.19)	0.92 (0.79, 1.07)

Adjusted for age (year), physical activity, current smoker, BMI, fruits, vegetables, cereal and protein sources.

## Discussion

In the present prospective cohort study in a large sample of Iranian adults, each additional increment in the frequency of total, animal and plant protein consumption was associated with lower odds of MetS. These results remained statistically significant even after adjustment for potential confounders and did not differ substantially between men and women. To the best of our knowledge, this study is among the first studies that prospectively examine the association of different dietary protein sources with incidence of MetS.

Due to rapid growth of MetS prevalent^34^, our findings, suggesting an inverse association between the frequency consumption of protein and MetS risk, can be considered to be important for public health. Despite a large number of studies examining dietary protein intake and various metabolic conditions, there is still debate in this context. Our findings regarding the inverse association of frequency of total and plant-sourced proteins consumption with MetS are consistent with previous observational studies^10,35^, though the association of animal protein with MetS remains controversial. While most of the studies found a direct association between animal protein and MetS^10,14,15^, one population-based study in Iranians demonstrated that higher dietary protein intake was associated with enhanced HDL-C levels, WC, and diastolic BP, while higher animal to plant protein ratio was associated with lower serum fasting glucose and WC^20^. The Melbourne Collaborative Cohort study demonstrated that higher plant protein consumption was associated with a decreased risk of MetS incidence, whereas higher total and animal protein intakes were associated with an increased risk^10^.

There are also several studies investigating the association between dietary protein intake and features of MetS. The Framingham Heart Offspring cohort suggested that higher protein intake was favorably associated with changes in SBP and unfavorably associated with changes in FBS, whereas plant protein was inversely associated with WC^36^. Some meta-analyses also demonstrated a favorable association between protein intake from both animal and plant sources and blood pressure, particularly when consumed in place of carbohydrate^37,38^. Higher protein intake may also lower body fat percentage^16^. In contrast with our findings, some cross sectional studies demonstrated a direct association between red and processed meat consumption and the risk of MetS^15,18^. These different findings might be explained by variations in the ethnicity, study design, sample size, statistical methods and potential confounders. The different association between dietary protein and MetS might be due to the different socioeconomic status. Unlike previous studies, our study was conducted in a developing and low-to-middle-income country. Furthermore, different methods used for processing and cooking of meats might explain further discrepancy.

The underlying mechanisms for the favorable relation between higher intake of dietary protein and MetS remain to be understood. Increased total protein intake results in a change in dietary macronutrients distribution^11^. In other words, the increase in dietary protein leads to an inevitably decrease in carbohydrate or fat consumption^11^. Hence, the beneficial effects of higher protein consumption may particularly depend on which macronutrients is replacing by protein^11^. When protein is consumed in place of carbohydrate, its potential effects depend on the type and content of dietary carbohydrate replaced by it^17^. The glycemic response is mostly influenced by amount and quality of carbohydrate in diet. Replacement of refined carbohydrate with protein may favorably affect glycemic response of diet by delaying gastric emptying and stimulating insulin secretion^17^. Carbohydrate, particularly refined type, makes a substantial contribution in Iranians’ diet. Moreover, the main features of dyslipidemia among Iranians are elevated triglyceride and decreased HDL-c concentrations, which are two components of MetS. Therefore, it is probable that an increase in protein intake to be associated with favorable changes in cardiometabolic profile. In addition, the frequency consumption of red and processed meats is not as much as higher the Western societies to exert detrimental effects. In support of our findings, earlier meta-analyses on red meat intake suggested just a little or no impact on the leading cardiometabolic outcomes, whose evidence is of low to very low of certainty^39,40^. Other possible explanations behind the favorable association between dietary protein and MetS might be related to increasing satiety, energy expenditure, reducing energy intake, fat mass and maintaining of lean body mass, and subsequently improving lipid profile and blood pressure^41–43^.

The strengths of our study include its prospective design, large sample size, long follow-up duration, a heterogeneous socioeconomic status population of Iranians, face to face interviews to collect data and repeated measurements. Some limitations need to be considered in the interpretation of our findings. First, we failed to examine portion sizes of various food items though dietary intakes were measured using a validated FFQ. As a result of this, it is not possible to exactly determine how much of protein intake is associated with lower risk of MetS. Second, measurement errors due to self-reported dietary data are another concern like any other epidemiological study which may result in the misclassification of participants. Third, although we adjusted our results for various confounders, the confounding effect of residual or bias related to unknown or unmeasured factors cannot be completely ruled out. Fourth, adjustment for some mediating factors like BMI in the last model may be an over adjustment and underestimates the true association. Fifth, although our earlier validation study revealed a significant correlation between animal protein intake estimated through FFQ with that of through a single 24 h dietary recall and a 2-day dietary record, it was not strong adequate and therefore our results should be interpreted cautiously and need to be confirmed by future studies.

In conclusion, our study provides evidence suggesting that subjects at high risk of MetS may benefit from increasing dietary intake of total protein, animal and plant protein. These associations were slightly stronger among men compared with women but did not considerably differ. Randomized clinical trials are required to confirm our findings.
